# Success Rates of Pulpotomies in Primary Molars Using Calcium Silicate-Based Materials: A Randomized Control Trial

**DOI:** 10.1155/2017/4059703

**Published:** 2017-10-31

**Authors:** Yeliz Guven, Sermin Dicle Aksakal, Nilufer Avcu, Gulcan Unsal, Elif Bahar Tuna, Oya Aktoren

**Affiliations:** Department of Pedodontics, Faculty of Dentistry, Istanbul University, Istanbul, Turkey

## Abstract

**Objective:**

The aim of this study was to evaluate and compare, both clinically and radiographically, the effects of calcium silicate-based materials (i.e., ProRoot MTA [PR-MTA], MTA-Plus [MTA-P], and Biodentine [BD]) and ferric sulfate [FS] in pulpotomy of primary molars.

**Materials and Methods:**

In this randomized clinical trial, 29 healthy 5- to 7-year-old children with at least four carious primary molars with no clinical or radiographic evidence of pulp degeneration were enrolled. The pulpotomy agents were assigned as follows: Group 1: BD; Group 2: MTA-P; Group 3: PR-MTA; and Group 4: FS. Clinical and radiographic evaluations were performed at 6, 12, and 24 months. Data were analyzed using chi-square tests.

**Results:**

Total success rates at 24 months were 82.75%, 86.2%, 93.1%, and 75.86%, respectively. No statistically significant differences in total success rates were observed among the groups at 6-, 12-, and 24-month follow-ups. When the groups were compared according to follow-up times, the success rates in each group did not vary significantly among the 6–12-month, 6–24-month, or 12–24-month periods (*p* > 0.05).

**Conclusion:**

Although the success rates of BD, MTA-P, MTA-PR, and FS did not differ significantly, calcium silicate-based materials appeared to be more appropriate than FS in clinical practice.

## 1. Introduction

Pulpotomy is the accepted treatment procedure for primary molars with exposed coronal pulps inflamed by bacteria due to caries, traumatic injury, or another iatrogenic cause [1]. The goal of pulpotomy is to remove infected coronal pulp tissue while preserving healthy radicular pulp, thereby promoting the integrity and retention of teeth until physiologic exfoliation [2]. However, pulpotomy is applicable only when the inflammation of the pulp is limited to the coronal tissue and the remaining root pulp is vital. Various pulpotomy medicaments aiming at devitalization, preservation, or regeneration of the remaining pulp tissue have been used to date. The ideal pulpotomy medicament would be bactericidal and biocompatible, promote the healing of the root pulp, and be compatible with the physiological process of root resorption [3]. Because such a medicament or technique with all of those features remains unavailable, however, given the lack of clear evidence supporting the superiority of any particular treatment method [4], research has continued to seek alternative pulpotomy agents that can provide better clinical efficacy without secondary effects.

To date, a range of pulpotomy medicaments have been used. Among them, formocresol (FC), a devitalizing agent with excellent bactericidal and fixative properties, has been the gold standard in pulpotomy for many years [5, 6]. However, despite its high success rate and popularity, its cytotoxicity, mutagenicity, and carcinogenicity have raised concerns in the medical community, especially after the International Agency for Research on Cancer classified formaldehyde as carcinogenic for humans in June 2004 [7].

Such concerns have prompted researchers to investigate alternative pulpotomy materials with better clinical efficacy and without secondary effects. Accordingly, various materials and techniques such as calcium hydroxide [8], ferric sulfate (FS) [9–11], mineral trioxide aggregate (MTA) [11–15], sodium hypochlorite [16], electrosurgery [17], and laser therapies [18, 19] have been used for pulpotomies. FS, a hemostatic agent used in dermatology and dentistry, has become a good replacement for FC in pulpotomies. Hemostasis occurs by agglutination of blood proteins and the agglutinated proteins form plugs that occlude the capillary orifices [3, 20]. FS provides clinical and radiographic outcomes similar to those of FC, as well as a nonaldehyde option for patients concerned about FC's toxic effects [20].

In recent years, a major advance in endodontics has been the development of MTA material with bioinductive and regenerative capabilities. Since 2001, MTA has served as a successful bioregenerative treatment alternative in pulpotomy of the primary molars [21]. Clinical trials have moreover shown that MTA has success rates similar or even superior to those of FC and FS [2]. Despite its advantages, including hard tissue formation and dentinogenesis in the pulp, MTA poses drawbacks such as a lengthy setting time, poor handling properties, and high cost [22, 23]. To overcome those shortcomings, researchers have modified the color, particle size, and formulation of MTA to yield commercial products launched with various brand names. MTA was introduced in 1993 [24] and received FDA approval in 1998 [25]. In 1999, ProRoot MTA (PR-MTA) (Dentsply, Tulsa Dental Specialties, TN, USA) became the first commercially available MTA product in the United States [26]. By contrast, MTA-Plus (MTA-P), though composed of ingredients similar to PR-MTAs, has a finer particle size—50% of particles are finer than 1 mm in diameter—which gives the material a higher specific surface area [27]. Even more recently, another generation of materials with properties similar to those of MTA has emerged in dentistry. Among them, Biodentine (BD) is a bioactive dentin substitute with a calcium silicate-based formulation that has attracted attention for its robust mechanical properties and high biocompatibility [28]. Indeed, such favorable properties, as several in vivo [29, 30] and in vitro [31, 32] investigations have shown, make BD a promising choice for pulpotomy of the primary molars. All these materials, including MTA products and Biodentine, are composed primarily of tricalcium silicate and named calcium silicate-based cements.

Although various materials have been proposed for the pulpotomy of primary molars, no reliable evidence suggests the superiority of one particular pulpotomy medicament and technique over the others [4]. Therefore, the purpose of this study was to evaluate and compare, both clinically and radiographically, the effects of calcium silicate-based materials (i.e., ProRoot MTA, MTA-P, and BD) and FS in the pulpotomy of primary molars. The null hypothesis was that there would be no difference between the success rates of four pulpotomy materials on the basis of clinical and radiographic criteria.

## 2. Materials and Methods

Participants were selected from patients attending the Department of Pediatric Dentistry at Istanbul University in Istanbul, Turkey. The clinical procedure, possible discomfort and risks, and possible benefits were explained to the parents or legal guardians of the participants, and written informed consent was obtained prior to investigation. The study protocol was approved by the Ethics Committee of the Istanbul Medical Faculty at Istanbul University (file number 2012/1728-1288). The study was registered at ClinicalTrials.gov (no. NCT03135626). The study design followed the CONSORT 2010 Statement of updated guidelines for reporting parallel group randomized trials [33]. A randomized, single-blind patient, split-mouth study design was used. Randomization was achieved by allocating each material equally to one of four quadrants. Two coauthors, both pediatric dentists, performed all pulpotomies. Although operators could not be blinded to group allocation due to obvious differences in materials, the dentists assessing the outcomes were blinded to group assignment. A power calculation indicated that a minimum of 28 teeth per group were required to detect a significant difference between groups when alpha risk is set at 0.05 and beta risk at 0.2 (*Zα* = 1.96,* Zβ* = 0.85). The sample size was increased to 38 subjects to compensate for potentially larger number of dropouts.

Patients were eligible for participation if they were 5–7 years old, healthy, and cooperative and had at least four carious primary molars, each in a different quadrant, that required a pulpotomy. The teeth of eligible patients were examined in detail both clinically and radiographically based on certain criteria. To be included in the study, teeth had to demonstrate deep caries, show the presence of two-thirds of the root length radiographically, and be restorable. The exclusion criteria were the presence of any clinical or radiographic evidence of pulp degeneration (e.g., spontaneous or nocturnal tooth pain, tenderness to percussion, pathological mobility, internal or external root resorption, swelling or fistula, widened periodontal ligament space, furcal or periapical radiolucency, and teeth requiring more than 3 min to achieve hemostasis during clinical procedure). Teeth without permanent successors were not included in the study.

The preoperative and control periapical radiographs were taken with a paralleling technique using a film holder (Rinn XCP; Dentsply, Elgin, IL). F Speed films (Kodak Insight, Eastman Kodak, Rochester, NY, USA) were used in conjunction with thyroid collars during the imaging sessions. The radiographs were processed in an automatic processor (Velopex® Intra-X Medivance Instruments, London, UK) with fresh chemicals. The radiographs were viewed and evaluated on a viewing box under optimum illumination. Pulpotomy procedures were performed after administering local anesthesia and rubber dam isolation. Caries removal and coronal access were achieved with a high-speed round diamond bur with ample water spray. Coronal pulp tissue was removed using a sharp, spoon-shaped excavator. Hemostasis was achieved by applying light pressure with a moistened sterile cotton pellet. If hemostasis was not achieved after 3 min, then the pulp tissue in the canal was assumed to be infected, and the patient was excluded from the study.

Each tooth was assigned to one of the four following groups depending on the pulpotomy medicament used.

*Group  1*. BD (Septodont, Saint Maur des Fosses, France) powder in the capsule was mixed with 5 drops of liquid in a triturator (4200 rpm) for 30 s. The mixture was placed in the pulp chamber and allowed to finish setting completely (i.e., for approximately 12 min). Permanent restoration was performed on the same session.

*Group  2*. MTA-P (Avalon Biomed Inc., Houston, Texas) was prepared by mixing 1 scoop of powder with 1 small drop of MTA-P gel until the desired putty-like consistency was obtained. A glass ionomer base was placed over the MTA.

*Group  3*. PR-MTA (PR-MTA) (Dentsply Tulsa Dental Specialties, Johnson City, TN, USA) was prepared by mixing 3 parts powder with 1 part water to obtain a putty-like consistency. This mixture was placed in the pulp chamber and condensed lightly with a moistened cotton pellet. A glass ionomer base was applied over the MTA.

*Group  4*. 20% FS solution was applied onto pulp stumps for 15 s; after rinsing with water, a ZOE base was placed. 

Following the pulpotomies, all teeth were restored with amalgam. Clinical and radiographic evaluations were performed at 6, 12, and 24 months. When a patient did not respond to contact from the researchers or missed an appointment, a follow-up examination was rescheduled. Clinical examinations were performed by an experienced pediatric dentist, but not the operator, blinded to the treatment groups. The pulpotomized tooth was considered to be clinical success if no swelling, pain, fistula, or pathologic mobility occurred. Radiographic examinations were performed by two experienced pediatric dentists, but neither the operators nor the clinical examiner, blinded to either the treatment groups or the clinical data. For the intraexaminer reliability of radiographic assessment, 24 radiographs (20% of the sample size) were reevaluated after 2 weeks. The intraexaminer kappa values of the two examiners were determined as 0.83 (strong) and 0.92 (almost perfect). The interexaminer kappa values for the two examiners at 6-, 12- and 24-month follow-ups were 0.90, 0.93, and 0.92, respectively, and the results were considered almost perfect. In the case of disagreement, the examiners discussed the case until they reached consensus. Teeth were considered to be a radiographic success if they showed no evidence of internal or external resorption or periradicular radiolucency. Pulp canal obliteration (PCO) was not regarded as a failure.

Statistical analysis was performed using the Statistical Package for the Social Sciences (SPSS, IBM Corporation, Version 21.0; Armonk, NY, USA) software. Chi-square tests were used to detect differences in the outcome measures in the four groups at each follow-up period. Differences in the performance of each material over time were gauged with McNemar's test. The level of significance was set at *p* < 0.05.

## 3. Results

**Figure 1:**
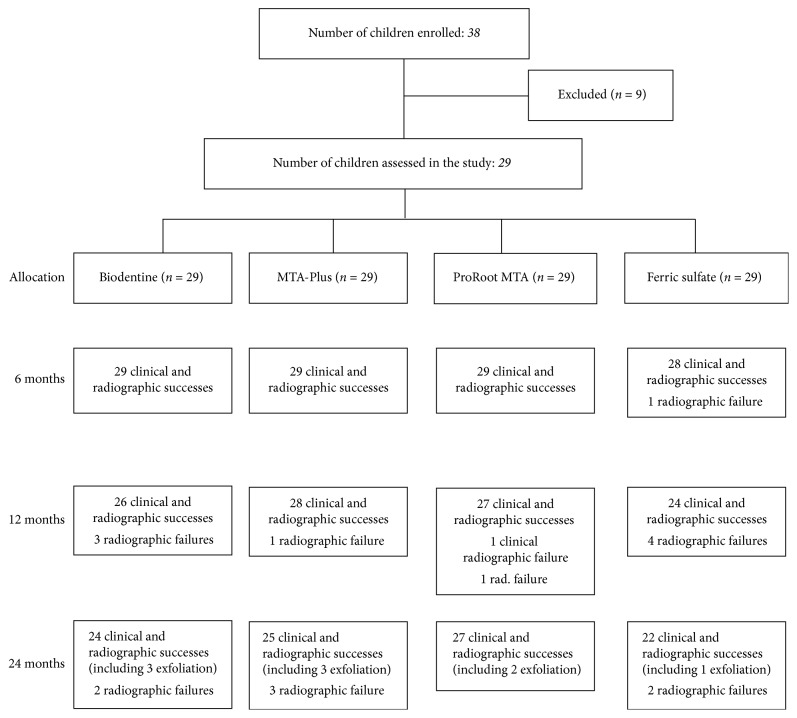
The flow of patients and pulpotomized teeth up to 24-month follow-up.

Thirty-eight children, each with at least four deep primary tooth caries on the first or second primary molars, were included in the study. Five children—two lost at 12-month follow-up and three at 24-month follow-up—were excluded from the study. Four children with failed restorations at one of the follow-ups were also excluded. In total, 116 pulpotomized primary molars in 29 children—19 males and 10 females—were evaluated statistically. At the beginning of treatment, children ranged in age from 5 to 7 years old, with a mean age of 5.86 ± 0.83 years. The flow of patients and pulpotomized teeth up to 24-month follow-up appears in Figure 1, and the distribution of the number of primary molars per pulpotomy material appears in Table 1. The total success rates of each group at the 6-, 12-, and 24-month follow-ups appear in Table 2.

Clinical and radiographical evaluation at 6 months showed 100% total success rates for the BD, MTA-P, and PR-MTA groups. One tooth in the FS group failed at 6 months due to periapical radiolucency and external root resorption, for a success rate of 96.55%.

At the 12-month follow-up, the sole clinical failure occurred in a tooth treated with PR-MTA showing abscess formation, pathologic mobility, and pain on percussion. Radiographic failures in each group appear in Figure 1. The total success rates at 12 months were 89.65%, 96.55%, 93.1%, and 82.75% for the BD, MTA-P, PR-MTA, and FS groups, respectively.

At the 24-month follow-up, no clinical failure was observed in the groups. In all, seven teeth demonstrated radiographic failure at 24 months (Figure 1). Total success rates of the BD, MTA-P, PR-MTA, and FS groups were 82.75%, 86.2%, 93.1%, and 75.86%, respectively.

No statistically significant differences in total success rates were observed among the groups at 6-, 12-, and 24-month follow-ups. When the groups were compared according to follow-up times, the success rates in each group did not vary significantly among the 6–12-month, 6–24-month, or 12–24-month periods. At 12 months, PCO was observed in two teeth treated with BD and one tooth treated with MTA-P; those teeth showed PCO at 24 months as well. At 24 months, four teeth in the MTA-P group, three in the BD group, and one in the PR-MTA group exhibited PCO. Three teeth in the BD and MTA-P groups, two in the PR-MTA group, and one in the FS group were naturally exfoliated at 24 months.

## 4. Discussion

This randomized clinical trial was conducted to examine and compare the effectiveness of pulpotomy in primary molars treated with calcium silicate-based materials—two MTA products (i.e., ProRoot MTA and MTA-P) and BD—and FS as the control material. Although most studies on pulpotomy materials have used FC as a control medicament [29], FS which provides outcomes similar to FC [9, 34] but offers a nonaldehyde option was chosen in the present study. The overall outcomes in this study revealed similarly high success rates, with no significant differences among the groups in terms of clinical and radiographic conditions.

MTA is increasingly recognized as the preferred choice of material for primary molar pulpotomies due to its superior biocompatibility and sealing ability, as well as its dentinogenic and osteogenic potential [5, 35]. MTA has been studied extensively as an alternative pulpotomy agent, and numerous clinical studies have shown that MTA pulpotomy has a high rate of success, both clinically and radiographically. Nevertheless, MTA pulpotomy has remained limited in clinical practice due to its high cost, long setting time, and difficult handling characteristics. MTA-P was developed to overcome those shortcomings by offering a lower cost, shorter setting time, and easier application. To date, however, no clinical studies have investigated the effectiveness of MTA-P as a pulpotomy agent in primary molars. In the present study, although MTA-P showed slightly a lower success rate (86.2%) than ProRoot MTA (93.1%) at 24 months, the difference was not statistically significant.

The success rates of MTA at 24 months in other evaluations of MTA as a pulpotomy material have ranged from 75% to 100% [36, 37] and usually exceeded 90% [11, 38–44]. The success rate of MTA pulpotomy observed in the present study thus corroborates results reported by other researchers.

A new bioactive material with dentin-like mechanical properties, BD has been used in primary molar pulpotomies due to its high biocompatibility and bioactivity, excellent sealing ability, short setting time, and ease of handling [29, 30, 45–47]. The 100% success rate of BD after 6 months in the present study concurs with the results of many other studies [29, 30, 45, 48]. At the 12-month follow-up with BD, Togaru et al., Rajasekharan et al., and Cuadros-Fernández et al. reported success rates of 95.5%, 96%, and 94.9%, respectively, all of which slightly exceed the rate of 89.65% in the current study [30, 45, 46]. No study on BD and MTA as pulpotomy materials found any statistically significant difference between them, which is consistent with the results of the present study [30, 45–47, 49]. Clinical studies examining BD as a pulpotomy material have been few and had relatively short follow-up times of 6–18 months. Sirohi et al. compared the 9-month success rates of BD and FS in their study and found that BD group (92%) had a higher success rate than FS group (80%), although this difference was not statistically significant [48]. Rajasekharan et al. reported that BD and ProRoot MTA had success rates of 94.4% and 90.9% at 18 months, respectively [46]. Juneja and Kulkarni reported success rates of BD and ProRoot MTA at 18 months as 86.7% and 100%, respectively [49]. In the present study, the 24-month success rates were 82.75% for the BD group and 93.1% for the ProRoot MTA group and this difference may be attributable to the longer follow-up period, different design, and different sample size.

A hemostatic and nontoxic agent, FS, has gained some popularity as a pulpotomy medicament for primary molars. In the present study, although no significant differences were observed among the materials, the lowest success rate was achieved with FS pulpotomy (75.86%). In a recent systematic review, Stringhini reported success rates of FS between 73.3% and 97.1% [17]. The success rates of FS in this study were similar to those observed by Sonmez et al. [37, 44, 50].

PCO is a common radiographic finding in pulpotomized teeth. Although some investigators [51] have considered PCO to constitute a radiographic failure because it demonstrates a deviation from normal pulp, most investigators [46, 52–54] agree to the contrary, since PCO results from the extensive activity of odontoblast-like cells that suggests retained vitality. In the present study, PCO was not regarded as a failure and was the most commonly observed radiographic finding. After 24 months, PCO was observed in 13.79%, 10.34%, and 3.45% of teeth in the BD, MTA-P, and ProRoot MTA groups, respectively. By contrast, no teeth treated with FS showed PCO. Kusum et al. observed PCO in 16% of teeth treated with BD and 20.0% of teeth treated with ProRoot MTA over a 9-month period [47]. Rajasekharan et al. revealed that their BD group showed more PCO than the ProRoot MTA group, which is consistent with the results of the present study [46]. Investigators who have tested MTA as a pulpotomy material over a 24-month period have reported PCO rates of 5.55% [40], 7.89% [39], 20% [11], 25% [41], and 26.7% [37]. In this study, PCO was observed in 10.34% of teeth treated with MTA-P and 3.45% of teeth treated with PR-MTA.

The most common radiographic finding in previous studies, internal resorption has been considered to indicate failure [11, 13, 21, 36], although some investigators have stipulated that it does not unless the process reached the root's outer surface [55, 56]. In the current study, internal resorption was considered to signify radiographic failure in all instances and occurred in 6.89% of the FS group and in 3.44% of both MTA-P and BD groups. The low prevalence of internal root resorption in calcium silicate-based materials in the present study corroborates the results of previous research. Watts and Paterson [57] suggested that zinc oxide eugenol can cause pulpal inflammation when directly applied to the pulp tissue, which explains internal resorption when that material is used as a base for pulpotomies. Most authors who observed a high rate of internal resorption in FS or FC pulpotomies have explained their results similarly [11]. However, our results indicate that FS pulpotomy did not exhibit a significant difference from the calcium silicate-based groups. Similar results were reported by Sonmez et al., who demonstrated that internal resorption rates did not significantly differ among their FC, FS, calcium hydroxide, and MTA groups [37].

The present study poses several limitations that warrant consideration. A major one relates to the use of amalgam as a coronal restoration. The unavailability of stainless steel crown due to financial constraints dictated treatment using amalgam restorations in all pulpotomized molars. To eliminate inconsistencies resulting from upper restorations, teeth with any extensive loss of structure requiring large restorations were excluded from the study. Additionally, a failed restoration during a follow-up period resulted in the exclusion of the tooth from the sample. Another limitation was the relatively high dropout rate (19.45%) of participants, likely due to the patient's attitude toward dental care. Usually, dental patients seek treatment when they feel pain; when treatment is complete and pain relieved, some patients do not return for follow-up visits.

## 5. Conclusion

This study found no statistically significant differences among pulpotomy techniques (*p* > 0.05); however, calcium silicate-based materials appeared to be clinically more appropriate than FS. Based on those results, calcium silicate-based materials can be considered to induce favorable effects on the reparative process during vital pulp therapy and to serve as alternatives to FS.

## Figures and Tables

**Table 1:** Distribution of the primary teeth according to the pulpotomy material.

	BD	MTA-P	ProRoot	FS	Total
Maxillary					
Primary 1st molar	5	2	9	2	18
Primary 2nd molar	9	6	5	4	24
Mandibular					
Primary 1st molar	7	9	7	12	35
Primary 2nd molar	8	12	8	11	39

Total	29	29	29	29	116

**Table 2:** Total success rates of the groups at 6-, 12-, and 24-month follow-ups.

Materials	Follow-up times					
	6 months *n* (%)	12 months *n* (%)	24 months *n* (%)	McNemar's 6/12 M *p*	McNemar's 12/24 *p*	McNemar's 6/24 *p*
BD	29 (100)	26 (89.65)	24 (82.75)	0.250	0.500	0.063
MTA-P	29 (100)	28 (96.55)	25 (86.2)	1.000	0.250	0.125
ProRoot	29 (100)	27 (93.1)	27 (93.1)	0.500	1.000	0.500
FS	28 (96.55)	24 (82.75)	22 (75.86)	0.250	0.500	0.063
*p*	0.388	0.534	0.491			
